# A rare case of polyarteritis nodosa associated with nontuberculous mycobacterial infection

**DOI:** 10.1002/ccr3.2414

**Published:** 2019-09-09

**Authors:** ShengYang Bertrand Lian, Yee Leng Teoh, Yong‐Kwang Tay

**Affiliations:** ^1^ Department of Dermatology Changi General Hospital Singapore Singapore

**Keywords:** cutaneous polyarteritis nodosa, infection, nontuberculous mycobacteria, polyarteritis nodosa

## Abstract

Clinicians should be aware that cutaneous PAN can present with significant extracutaneous and constitutional symptoms which make it hard to differentiate from systemic PAN. The condition can also rarely be associated with NTM infections.

## 1 CASE REPORT

A 49‐year‐old Chinese woman with a history of treated pulmonary tuberculosis in 2005 presented in June 2013 with a two‐month history of productive cough and weight loss. On examination, she was cachectic with left lung basal crackles audible on auscultation. Computed tomographic (CT) scan of the thorax revealed lingual consolidation and prominent hilar lymph nodes. Bronchoscopic examination of the lung tissues was unremarkable. Acid fast bacilli cultures of the sputum and bronchoalveolar lavage specimens were positive for *Mycobacterium fortuitum* and *Mycobacterium abscessus*. She was treated with oral clarithromycin, intravenous amikacin, and cefotaxime based on the antibiotic susceptibility testing, leading to the resolution of respiratory symptoms within 3 weeks.

**Figure 1:**
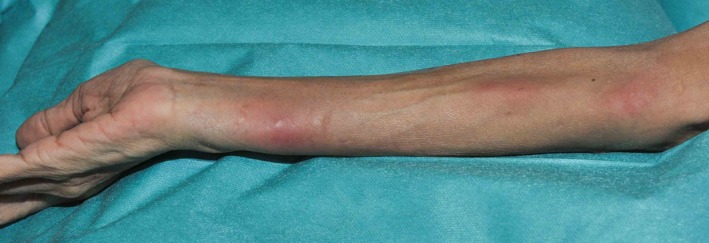
Erythematous subcutaneous nodules in a sporotrichoid pattern on her right forearm

**Figure 2:**
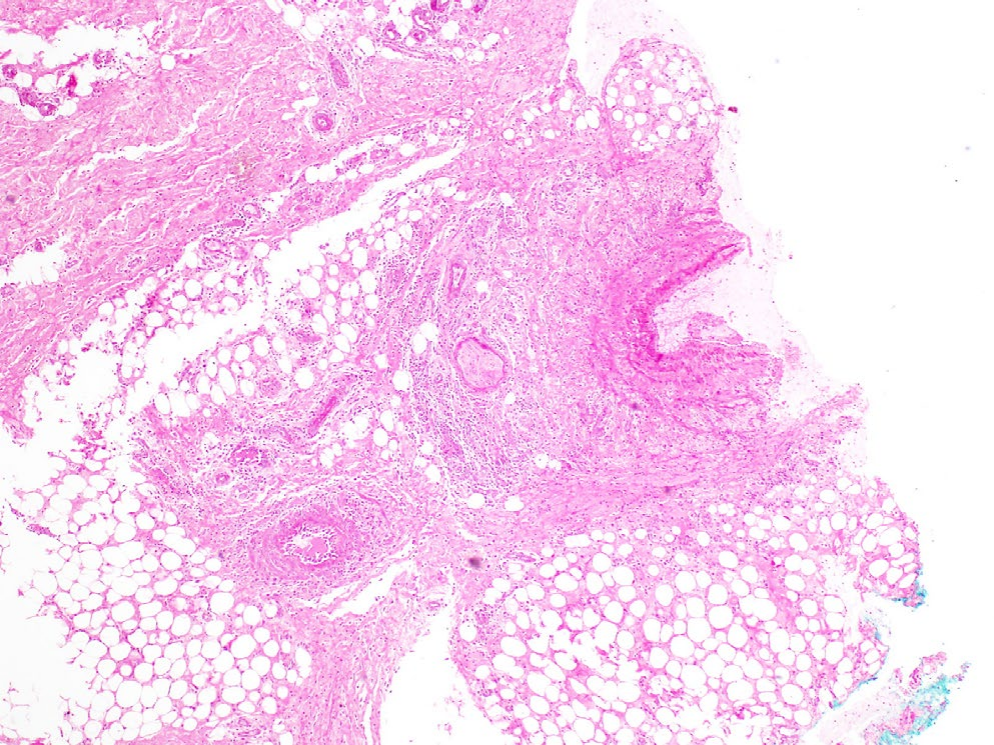
Vasculitis of medium‐sized blood vessels in the lower dermis with no involvement of the small vessels of the superficial dermis

In August 2013, she developed a persistent myalgia, paresthesia on her distal upper limbs and sustained a weight loss of 4kg over 4 weeks. She did not have fever, chills, or rigors. On examination, several tender, erythematous subcutaneous nodules were present on her right forearm and hand (Figure 1). There was no evidence of livedo reticularis. An excision biopsy of her right forearm nodule revealed vasculitis of medium‐sized blood vessels in the lower dermis with no involvement of the small vessels of the superficial dermis (Figure 2). There were no granulomas or caseating necrosis present. She was normotensive with laboratory investigations including complete blood counts, serum urea, and creatinine levels that were normal. Hepatitis B screen and antinuclear cytoplasmic antibodies (ANCA) were also negative. Nerve conduction studies of the upper limbs were unremarkable. CT angiography of the abdomen revealed a 0.9cm by 0.8cm saccular aneurysm of the right renal artery.

The presence of medium vessel vasculitis on histology with a negative ANCA was deemed consistent with polyarteritis nodosa (PAN) as defined by the Chapel Hill Consensus Conference.^1^ However, with only features of persistent myalgia and consistent histology, she did not convincingly fulfill the criteria for systemic PAN as defined by the American College of Rheumatology.^2^ The weight loss was confounded by concomitant nontuberculous mycobacterial infection, and the single renal artery aneurysm was not typical of microaneurysms seen in systemic PAN.^3^ Nonetheless, in view of her marked constitutional symptoms, she was treated as for systemic PAN.

She was started on oral prednisolone 30mg once daily with good resolution of her skin lesions and systemic symptoms within three weeks. Mycophenolate mofetil was commenced as a steroid‐sparing agent while oral prednisolone gradually tapered. To date, she has no signs or symptoms to suggest recurrence of PAN and is currently on oral prednisolone 7.5mg once daily and mycophenolate mofetil 500mg twice a day.

Systemic PAN is a multisystem vasculitis of the medium‐sized arteries. It is distinguished from cutaneous PAN by the presence of systemic manifestations.^4^, ^5^ There is ongoing debate on whether they constitute a single disease spectrum or two distinct disease entities. While authors have attempted to establish diagnostic criteria for cutaneous PAN,^6^ there has yet to be a universally agreed consensus. This may partly be due to the prevalence of extracutaneous manifestations in patients with cutaneous PAN.^4^, ^7^ While our patient did not demonstrate definite systemic manifestations, she presented with significant constitutional and extracutaneous symptoms. Cases such as this highlight the difficulty faced by clinicians in distinguishing between systemic and cutaneous PAN.

The presence of multiple nontuberculous mycobacterial species in pulmonary infection is also uncommon, and its association with PAN is rare.^8^ While the pathophysiology of PAN is unknown, we theorize that a delayed‐type hypersensitivity response may be responsible for its onset after mycobacterial infection. Dendritic cells facilitate antigenic exposure of naïve CD4 + T lymphocytes with activation and release of inflammatory cytokines. This inflammatory cascade in turn causes further activation of macrophages and vessel wall damage.^9^ It is plausible that a nontuberculous mycobacterial antigen may be responsible for triggering the onset of delayed‐type hypersensitivity in this form of vasculitis.

## CONFLICT OF INTEREST

None declared.

## AUTHOR CONTRIBUTION

SYBL: involved in manuscript research and preparation; YLT and YKT: involved in the care of the patient and critical review of the manuscript.
